# Aphid adaptation to plant secondary metabolites: adaptive mechanism of resistance evolution and future prospects

**DOI:** 10.1093/hr/uhaf269

**Published:** 2025-09-28

**Authors:** Muhammad Farhan, Jilong Pan, Jun Zhao, Hanjing Yang, Shuai Zhang

**Affiliations:** College of Plant Protection, Yangzhou University, Yangzhou 225009, Jiangsu, China; College of Plant Protection, Yangzhou University, Yangzhou 225009, Jiangsu, China; College of Plant Protection, Yangzhou University, Yangzhou 225009, Jiangsu, China; College of Plant Protection, Yangzhou University, Yangzhou 225009, Jiangsu, China; College of Plant Protection, Yangzhou University, Yangzhou 225009, Jiangsu, China

## Abstract

Aphids are demonstrated to be voracious phloem feeders, among the most damaging insect pests, due to their capacity to decrease crop production and vector plant viruses. Plant secondary metabolites (PSMs) comprise an essential element of plant protection, which in most cases deters and affects aphid performance. Nonetheless, aphids have developed various resistance mechanisms to counteract these chemicals. This review provides an extensive overview of the biological and molecular adaptations that aphids employ to counteract PSMs, including enzymatic detoxification, antioxidant defense, sequestration, behavioral response shifts, suppression of plant defense mechanisms by symbionts, and manipulation of host signaling pathways by effector proteins. We also described the suppression of the defense pathways by aphid-associated viruses, which further complicates plant–aphid interactions. Although significant insights have been gained about each of the individual mechanisms, research gaps remain, particularly in the functional confirmation of detox genes, the communication interactions of the symbionts, and whether sequestration could play an ecological role across species. Intensive efforts involving molecular-based breeding of horticultural crops, as well as traditional breeding with wild relatives highly endowed with aphid-resistant PSM traits, should be employed in the future to provide sustainable crop protection. New technologies in crop genomics, the identification of effectors, and microbiome research promise the development of resistant cultivars that are not only resistant to aphids but also prevent the spread of disease by their vectors. Together, all this knowledge has the potential to produce high-yielding crops that are resistant to aphids and to implement sustainable farming practices.

## Introduction

Aphids are small (<7 mm), soft-bodied insects that cause severe agricultural losses worldwide by feeding directly on plants and transmitting numerous plant viruses. Nearly, 5696 species are currently recognized as alive**,** according to the most recent update of the Aphid Species File, with approximately 250 species reported as major pests of agricultural and horticultural crops worldwide [1, 2]. Mostly, aphids live under the leaves in the form of colonies or on the soft parts of terminal shoots, suck plant sap, and secrete an extensive volume of liquid that contains sugar called honeydew. This sugary material then turns into a black fungus called sooty mold that becomes the source of food for bees, ants, and may indirectly affect parasitoid wasps by altering their interactions through ant activity [3]. Still, it reduces the photosynthetic area available to plants, resulting in reduced growth and lower production. Moreover, out of the total insect-transmitted viruses in plants, 50% are transmitted by aphids [4]. Aphids have different morphs, like alate and aptera [5]. Due to the voracious feeding behaviour of aphids, they cause millions of dollars in losses annually worldwide [6]. Aphids have evolved a complex reproductive mode, including sexual males, asexual females, and egg-laying females. However, reproduction through asexual females is called parthenogenesis, which enables their survival across different seasons. Parthenogenesis is a rapid mode of reproduction that typically occurs within 10 days, across many world regions, especially in temperate areas, allowing populations to increase rapidly [2, 7].

Effective management of aphid populations is crucial for minimizing economic losses resulting from their rapid and diverse reproductive modes in agricultural and horticultural crops. However, this review focuses on aphid adaptation to PSMs. Still, it is essential to recognize that aphid infestations in greenhouse systems are actively controlled with the help of the integrated pest management (IPM) methods involving the release of biological control organisms, e.g. in a greenhouse experiment, *Aphidius colemani* has been demonstrated to be a potent suppressor of *Myzus persicae* and other aphids [8]. Biocontrol based on greenhouses has also proven successful in managing aphids in banker plant systems and ornamental greenhouses, utilizing these biocontrol methods [9]. Interestingly, it is unfortunate that growers continue to rely on synthetic insecticides to combat aphids. Excessive utilization of synthetic insecticides by farmers has led to resistance in aphids. Nearly, 20 major species of aphids have evolved resistance to at least one insecticide worldwide, posing a significant threat to the control of other aphid species. According to 2025 data, the Arthropod Pesticide Resistance Database (APRD) reported 1218 cases of insecticide resistance in aphids, linked to 30 aphid species, with most of them being concentrated in economically important species, *M. persicae* (522 cases) and *Aphis gossypii* (438 cases) [10].

In agricultural systems, two long-term, large-scale forces have determined the evolution of aphid adaptation, specifically (i) large-scale insecticide application, which has selected repeatedly to promote metabolic and target-site resistance (the most notable example being *M. persicae*), and (ii) the expression of resistant/tolerant cultivars, which have likewise chosen virulent aphid biotypes. The first cases of insecticide resistance in *M. persicae* were reported in the 1950s, and they were followed by the description of several resistance mechanisms and virulent biotypes able to overcome resistant cultivars in the 1970s–80s in Europe and other parts of the world [11, 12]. Genomic and transcriptomic studies have provided the molecular mechanisms of such adaptations since the 2000s, including copy-number variation of detoxification genes and the high rate at which resistance alleles disseminate [2]. We have reviewed major field crops, including wheat, maize, and brassica, as well as horticultural crops grown in greenhouses, such as tomatoes, cucumbers, and melons, where interactions with plant secondary metabolites have been well studied. The need to use biochemicals promotes IPM strategies for sustainable agricultural practices. In this regard, plants have evolved defensive PSMs, which have been utilized to protect them against both biotic and abiotic stresses. These compounds do not play a role in the growth and development of the plant, unlike primary metabolites; however, they are instrumental in generating resistance to insect pests. Some researchers have indicated that aphids are repelled, deterred from feeding, and even killed by these compounds, thereby offering a potential environmentally friendly approach to aphid management [13]. Nonetheless, the efficiency of PSMs is limited because, similar to synthetic insecticides, aphids can adapt to them to resist the action of various PSMs and overcome their toxic effects through a range of mechanisms, including the production of degradation enzymes, microbial symbiotes, salivary proteins, vectoring viruses, and behavior changes [14]. Numerous studies have specified that herbivores are capable of quickly evolving to accommodate the PSMs and even benefit by finding their host plants and sequestering plant toxins to defend themselves against predators. This battle between aphids and the plants’ defense mechanisms illustrates the intricacy of the interaction between aphids and plants, as well as the deficiency of the PSM in pest control. In this regard, a detailed analysis of the aphid reaction to some PSMs based on omics technologies is necessary to comprehend the molecular pathways that explain the mechanism on how the aphids avoid such defensive compounds and to develop varieties that are resistant against aphid by, among others, quantitative trait loci (QTL) mapping and genome-wide association studies (GWAS). For example, transcriptome analysis and QTL mapping have successfully identified the aphid-resistant genes in cotton, maize, cucumber, and soybean [15, 16].

In this review, we briefly highlight potential of PSMs as biopesticides against different aphid species, the development of resistance mechanism in aphids against defensive metabolites of plants that enable aphids to adapt under these compounds stress under current molecular discoveries that enhance our understandings of aphid–plant interaction, and breeding or engineering new resistant plant varieties rich in PSMs that provide future insight into IPM and research and can minimize the use of synthetic pesticides. Innovative research aims to identify the exact molecular mechanisms by which aphids adapt to crop plants, thereby facilitating the development of effective strategies to combat aphid resistance.

## Plant secondary metabolites as defensive agents and their dynamic regulation

To compete with various insect pests, plants produce four types of defensive chemical substances, known as PSMs, including terpenoids, phenolic compounds, N-containing compounds, and S-containing compounds [17]. These chemicals have proven their effectiveness in natural insecticides for managing aphids and other insect pests, as they repel pests, kill them, and attract their natural enemies [13].

Structurally, the leading PSM group is terpenes, which are indirectly involved in plant defense (including direct toxicity, anti-feeding, and oviposition deterrence of herbivores). Terpenes are primarily categorized into sesquiterpenoids, monoterpenoids, and triterpenoids, which are typically the dominant compounds of essential oils and, in some instances, are used physiologically and ecologically in the plant kingdom, such as being used as allelopathy agents and attracting pollinators by a release of volatiles [18]. For example, Cucurbitacin B is a triterpenoid that is effective against *A. gossypii* and exhibits both toxic and antifeedant properties [19]. Farnesol is a plant-derived sesquiterpenoid, exhibiting insecticidal activity against *M. persicae* and antimicrobial properties, as well as flavoring properties [20]. In the same manner, d-Limonene is part of the monoterpenoids that exhibit aphicidal activity against *A. gossypii* and *M. persicae* [21].

Phenols constitute a heterogeneous category of PSMs, based on their chemical structure, which features one aromatic ring. Notably, simple compounds are also present in phenols, such as flavonoids, stilbenes, tannins, and lignins as well, which are polymers derived from phenylpropanoid units [22]. They also contribute to the defense of plants against insect attacks; for example, tannic acid has shown antifeedant effects against *A. gossypii* and *M. persicae* [23,24]. Moreover, quercetin, a flavonoid, exhibited aphicidal activity against *Macrosiphum rosae* [25]. Similarly, alkaloids, such as caffeine, colchicine, nicotine, morphine, and ergolines, are N-containing PSMs. Alkaloids pose adverse effects on insects by interrupting nerve transmission, as well as disturbing cytoskeletal and cell membrane structure, DNA replication, enzyme activity, and protein synthesis [22]. These compounds, for example, capsaicin, also showed aphicidal properties against *M. persicae* and *A. gossypii* [26]. However, S-containing defensive compounds in plants are mainly of two types, such as alliins and glucosinolates, and are relatively involved in defense against herbivores [18]. Camalexin and glucosinolates have been reported as antifeedants against *Diuraphis noxia* and *M. persicae* [27]. We extensively described the insecticidal mechanism of numerous PSMs in our previously published review [13].

In addition to structural diversity and insecticidal mode of action, the functional activity of PSMs is strictly controlled by spatial, temporal, and inducible patterns within the plant system. The production mechanism of PSMs is complicated and herbivore specific, as these compounds are only released in the plant’s biosystem or into the environment upon herbivore attack, which helps plants conserve energy. Plant hormones, including salicylic acid (SA) and jasmonic acid (JA), control this triggered mechanism of resistance in plants. The most considered oxylipin in plant defense is JA, which is highly induced by insect herbivory. However, many phloem-feeding insects induce SA-dependent defenses; some phloem feeders, such as the *Bemisia tabaci* [28], *Nilaparvata lugens* [29], and *Sogatella furcifera* [30], induce JA-dependent defenses, whereas chewing insects induce JA-dependent defenses [31]. Different species of aphids pre-infesting can also differentially modulate changes in the plant’s hormonal defense mechanisms; for instance, *A. spiraecola* increased JA-dependent responses in citrus, which reduced the performance *of Diaphorina citri*. On the other hand, *Aphis citricidus* inhibited JA signaling, leading to improved feeding and reproduction of the psyllids [32]. The coevolutionary interaction between aphids and plants is maintained by selective pressures resulting from plant chemical defenses, which lead to specific adaptations in some aphid species to their preferred host plants. For example, *M. persicae* exhibited enhanced detoxification enzyme activity in *Arabidopsis thaliana* bik1 mutants, and the aphids were able to survive oxidative stress [33]. In this dynamic of an evolutionary arms race between plants and insect herbivores, these phloem-sucking aphids can overcome the plant defense mechanism to maximize their fitness for survival. These patterns of complexity in aphid–plant interactions may increase the evolutionary pressure that leads to the development of resistance against plant defenses.

## Resistance mechanism of aphids against plant defense

Throughout evolutionary history, aphids and their host plants have evolved to establish complex interactions, where either organism affects the other in its characteristics and adaptations. As plants produce different kinds of defensive phytochemicals in response to aphid attack, aphids counterattack on PSMs by various means, including detoxifying them with enzymes and behavioral adaptations. In the following, we briefly describe the evolution of resistance mechanisms in aphids against PSMs.

## Mechanism of detoxifying enzymes

**Figure 1:**
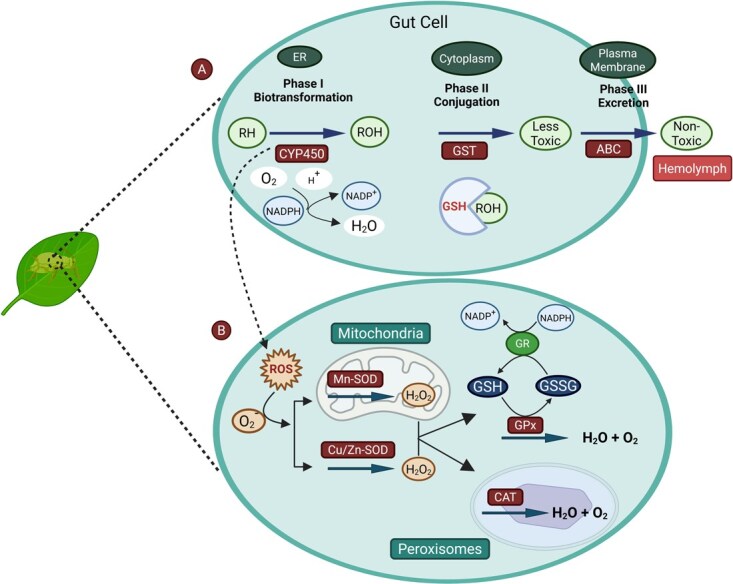
Mechanism of detoxifying enzymes. (A) In case of detoxification enzymes, phase I involves the biotransformation of toxic PSMs (RH) into hydrophilic compounds with the help of CYP450 enzyme in ER (Endoplasmic Reticulum), which are more polar (ROH) by introducing reactive oxygen molecules and NADPH, which release H_2_O and NADP^+^. Phase II is called conjugation, which takes place in the cytoplasm, where these ROHs conjugate with glutathione (GSH), facilitated by enzymes such as GSTs, COEs, and UGTs, to make them water-soluble and less toxic. Phase III, which takes place in the plasma membrane, involves the excretion of conjugated compounds from the cell through ABC transporters to minimize cellular toxicity. The ABC transporters located at the plasma membrane release metabolites to the gut lumen or hemolymph, which are later excreted. [35]. (B) The dotted arrow in Phase I (CYP450 activity) to panel B depicts that antioxidant enzymes further neutralize the ROS produced during detoxification reactions. Different ROS, like O_2_^−^, are produced in the aphid cell, which causes oxidative stress. To counter them, SOD (Mn-SOD in mitochondrial matrix and Cu/Zn-SOD in cytosol) converts O₂^−^ into H₂O₂. However, CAT is involved in the breakdown of H₂O₂ into water and oxygen molecules in peroxisomes [38]. Similarly, GPx also converts toxic H₂O₂ into water and oxygen by reducing GSH into GSSG (oxidized glutathione), where GR (glutathione reductase) converts GSSG back into GSH by using NADPH molecule [34].

Insects have evolved mainly two types of enzymes, namely detoxifying and antioxidant enzymes, which perform distinct but overlapping functions to counteract PSMs. Detox enzymes, such as cytochrome P450s (CYPs), UDP-glycosyltransferases (UGTs), and glutathione S-transferases (GSTs), neutralize the defensive effect of PSMs, enabling successful adaptation to host plants. These enzymes utilize metabolic pathways that couple, alter, and excrete toxic compounds, thereby neutralizing the plant’s defensive compounds. However, antioxidant enzymes, such as catalase (CAT), glutathione peroxidases (GPX), and superoxide dismutase (SOD), detoxify reactive oxygen species (ROS) produced during environmental exposure or metabolic stress, thereby averting oxidative damage to cellular structures. Although GSTs play both roles as detoxification agents and as a form of antioxidant protection to the body. The facts reveal that detoxification enzymes focus on the metabolism of xenobiotics, whereas the antioxidant enzyme groups focus on scavenging ROS. In summary, such systems of enzymes enable insects to withstand poisonous challenges and maintain cellular homeostasis [34]. In general, the detoxification of PSMs and other exogenous, adverse xenobiotics in aphids has been shown to occur in three phases of metabolism. The phase I reaction involves the cytochrome P450 monooxygenase, which incorporates a reactive or polarizing group into lipophilic toxins, thereby reducing their biological activity and rendering them more accessible to further metabolism. In phase II, the conjugation of these modified compounds with water-soluble molecules (including glutathione or sugars, etc.) is an enzymatic process. It is catalyzed by enzymes, such as carboxylesterases (COEs), GSTs, and even UGTs. Finally, in phase III, the conjugated metabolites are moved out of the cell through ATP-binding cassette (ABC) transporters and other membrane-bound pathways, thus reducing cellular toxicity [35] (Fig. 1A). As an example, gramine and gallic acid (PSMs) were found to cause up-regulation of the particular GST and SaveGST1 genes in *Sitobion avenae* and thus proved their potential to be detoxified [36]. In *A. gossypii*, the plant-derived compounds, epigallocatechin gallate and cucurbitacin B, triggered a high induction of the gene *AgoCYP6CY19*, which is a candidate cytochrome P450 gene involved in plant allelochemical detoxification [37]. The gene encoding an ABC transporter in *Acyrthosiphon pisum*, *ApABCG17*, was upregulated in response to tannic acid exposure. In contrast, RNAi knockdown of the relevant gene increased the aphid susceptibility to tannic acid, thereby indicating a significant role in PSM detoxification [24]. However, minimal information is available to confirm the functional mechanism of each detox gene, creating a critical research gap.

Antioxidant enzyme systems were initially evolved to detoxify the reactive oxygen species (ROS) produced under normal respiration in aerobic organisms. Exposure to PSMs and abiotic stressors may additionally induce or upregulate these enzymes in insect herbivores and increase their power to scavenge and counter oxidative stress [39,40]. The vital enzyme in this defense mechanism is SOD, which catalyzes the dismutation of superoxide radicals to hydrogen peroxide and molecular oxygen. It occurs in various isoforms, specifically Cu/Zn-SOD in the cytosol and Mn-SOD in the mitochondrial matrix, which protect different cellular compartments. An enzyme called CAT plays a crucial role in the detoxification of hydrogen peroxide, breaking it down into water and oxygen. Consequently, catalase prevents the toxic effects of accumulated hydrogen peroxide [38]. GPx additionally increases the antioxidant power by reducing hydrogen peroxide and organic hydroperoxides to nontoxic substances with the help of reduced glutathione (GSH) as an electron donor. This reaction neutralizes peroxides and aids in achieving redox homeostasis in cells as well [34] (Fig. 1B). Oxidative stress caused changes in H₂O₂ and activities of SOD and CAT in *M. persicae* and *Rhopalosiphum padi* in response to essential oils consisting of PSMs [41]. The SOD and CAT activities in aphid tissues were also found to be drastically different when the host plants were switched, especially those with bountiful allelochemicals, indicating a response to PSM-instigated oxidative stress via detoxification [42]. Increased activity of antioxidant enzymes was found in *M. persicae* when it was exposed to host plants like cumin and coriander, which are coupled with the production of pro-oxidant compounds, i.e. alkaloids like furanocoumarins [43]. At high levels of gramine and catechol, the antioxidant enzyme CAT was inhibited in *Sitobion avenae*, whereas low levels of the same promoted activity, thus denoting a dose-dependent effect of PSM [44].

Though some studies point to antioxidant enzymes in aphid defense against oxidative damage, there is little evidence that directly implicates specific secondary metabolites in plants in the production of ROS and antioxidant defenses of aphids, which is more scattered. The above examples and mechanisms demonstrate that aphids are strengthening their resistance to adapt against PSMs through the use of detoxifying and antioxidant enzymes [34,42].

## Symbiotic relationship

The presence of symbiotic bacteria is familiar in aphids and other insects, categorized into two types: obligate symbionts and facultative symbionts. Symbiotic bacteria have positive outcomes on the physical health of the host insects [45]. Bacterial symbionts, which suppress plant resistance or overexpress detoxifying enzymes, help improve the host’s adaptation more effectively [46]. Some symbionts can also detoxify various inorganic and organic compounds, including insecticides and PSMs. For instance, infection of *Regiella insecticola* improved the performance of *Sitobion avenae* on wheat cultivars against a plant toxic compound, benzoxazinoid DIMBOA [47]. Similarly, gut symbiont *Sphingomonas* in *A. gossypii* metabolizes imidacloprid through hydroxylation and nitroreduction [48]. This skill is remarkable, as it benefits certain insects, enabling them to colonize low-competitive ecological niches [49]. Caffeine is a deadly alkaloid for some insect pest species found in coffee beans. However, *Hypothenemus hampei* (coffee berry borer) can complete its whole life cycle on the same plant with the help of microbiota [50]. Similarly, a species of guava fruit fly (*Anastrepha striata*) successfully adapts to guava because its symbiotic Komagataeibacter can efficiently detoxify polyphenols and tannins [51]. Many other insect species, such as the mountain pine beetle, pine weevil, camellia weevil, olive fly, etc., have been studied to confirm the role of gut symbionts in detoxifying phytotoxins [52] (Table 1). There have been nine different types of facultative symbionts defined, of both vertical and horizontal transmission modes, with effects on a variety of ecological traits. Some of these include *Serratia*, *Hamiltonella*, *Rickettsia*, *Regiella*, *Arsenophonus,* and *Spiroplasma*; however, *Buchnera* is an obligate symbiont found in all aphid species, which performs specific nutritional functions [53]. The symbiotic bacteria in aphids increase plant infestation. *Serratia*-protective aphids suppress the generation of ROS in plants during feeding. They further inhibit the defense cues related to JA and SA, thereby weakening the plant’s resistance to aphids [54]. The secondary symbiont *Hamiltonella defensa* in *Sitobion miscanthi* enhances aphid fitness and undermines wheat defenses by downregulating SA and JA signaling. This interference is mirrored by lower activity of protective enzymes, such as peroxidases and polyphenol oxidase, thereby providing evidence of favoring herbivore adaptation by manipulating plant defense [55]. As per our knowledge, a similar study on the suppression of JA and SA signaling has not yet been conducted in other species of aphids; however, *H. defensa* has been demonstrated to improve the feeding behavior of *R. padi*, and *A. pisum* showed protection against parasitoids [56, 57].

**Table 1:** Symbiont-mediated adaptations in some insect species against PSMs and plant defense signals.

**Insect species**	**Host plants**	**Symbionts**	**Role of symbionts**	**References**
Olive fruit fly (*Bactrocera oleae*)	Olive	*Candidatus* Erwinia dacicola	Symbiont-mediated counter of oleuropein (phenolic glycoside) in unripe olives	[58]
Camellia weevil (*Curculio chinensis*)	Tea oil camellia	*Acinetobacter* sp. AS23	Degrades tea saponins (Triterpenoid saponins)	[59]
Pine weevil (*Hylobius abietis*)	Conifers	Gut bacteria (*dit* gene cluster)	Degrades diterpene acids	[60]
Mountain pine beetle (*Dendroctonus ponderosae*)	Pines	*Rahnella*, *Burkholderia, Pseudomonas,* and *Serratia*	Degrades terpenes	[61]
Cabbage stem flea beetle (*Psylliodes chrysocephala*)	Oilseed rape	Gut bacteria of the *Pantoea* genus	Degrades isothiocyanates	[62]
Colorado potato beetle (*Leptinotarsa decemlineata*)	Tomato, Potato	*Pseudomonas*, *Enterobacter*, and S*tenotrophomonas*	Suppresses plant defense signals	[63]
*B. tabaci*	Cotton	*Rickettsia*	Inhibits JA signaling pathways and activates SA	[64]
*Bemisia tabaci*	Cotton	*Cardinium*	Inhibits plant defense response	[65]
*A. pisum*	*Medicago truncatula*	*S. symbiotica*	Weakens plant defense response by inhibiting Ca^2+^ rise and ROS accumulation	[66]

Considering the above examples of symbiotic detoxification of PSMs by other insects, such as the coffee berry borer and the guava fruit fly, we can conclude that symbionts can play a role in detoxifying various toxic metabolites; however, substantial evidence supporting this claim remains insufficient in aphids.

## Effectors mediate adaptation

**Figure 2:**
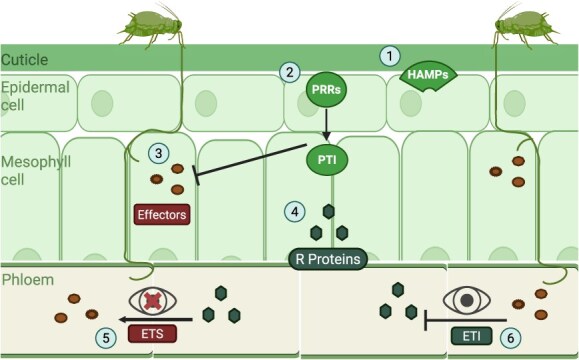
Mechanism of effector-mediated adaptation. (1) On aphid infestation, plants can identify HAMPs (Herbivore-associated molecular patterns) by PRRs (2), which trigger PTI. (3) To counter this defense, aphids produce salivary effector proteins that suppress the plant’s PTI. (4) However, plants also have the counter R proteins, but when these resistant genes fail to recognize aphids’ effector proteins, it leads to ETS (5). (6) In contrast, when these proteins successfully recognize effectors, it leads to ETI [68].

The phloem-feeding aphids release effector proteins in their saliva that can hijack the plant defense. Such effectors are crucial in the colonization of a host plant by the aphid. This is because the effectors can suppress the host’s defense responses, allowing aphids to generate a favorable environment for feeding and reproduction [54]. According to the zigzag model, which demonstrates the genetic interaction between insects and plants, plants recognize different types of molecules associated with herbivores, such as effector molecules, by using their resistant genes or proteins (R). Similarly, insects can adapt to these resistant proteins in plants through effectors in the zigzag hypothesis. The process of insect feeding activates plant defense signals mediated by transmembrane pattern-recognition receptors (PRRs), resulting in the activation of PAMP-triggered immunity (PTI). Aphids overcome PTI in plants by interacting with plant cell proteins through their effectors, which alter cellular activities and processes. In situations where plant immune receptors do not recognize the aphid effectors, this may result in effector-triggered susceptibility (ETS), allowing the invading effectors to become adapted to the host cell’s processes. However, the R proteins can also detect insect effectors to attack them leading to effector triggered immunity (ETI) [67,68] (Fig. 2). But in its turn, the aphids may evolve new effectors to recognize these R proteins to resist ETI [69]; it can become a cycle of adaptation and counter-adaptation characterized by the zig-zag pattern. Soybean aphids have been observed to have an adaptation that counters the R genes in soybean plants. It is believed that specific effectors are involved in this mechanism; however, the exact nature of this particular mechanism remains unclear and requires further research [70]. Nevertheless, the effector genes of the soybean aphid have been observed to be downregulated in response to host plant resistance (HPR) [71]. Sm9723, Sm10, and SmC002 are salivary effectors proteins found in the grain aphid *Sitobion miscanthi*, which play a vital role in aphid survival on wheat. It is strongly expressed in aphid salivary glands when they feed and inhibits the defense reactions in plants [72, 73]. Mp10 and Mp42 effectors are found in the *M. persicae* and also induce plant defense in *Nicotiana benthamiana* by performing various activities. Although their exact modes of action remain to be determined, they have been observed to be localized in the host cells and to impair plant defense. However, Mp10 was recorded as activating the JA and SA pathways and suppressing the expression of Agrobacterium, exhibiting specific immune mechanisms [74]. In addition to inhibiting the R-gene recognition process, some effectors impair the plant’s calcium (Ca2+) signaling, a primary line of defense, which in turn suppresses PSMs production. For example, different salivary proteins of *M. persicae*, including MP1 and MP55, have been shown to inhibit the plant’s defensive mechanisms [75]. The injection of salivary proteins by the aphid into the phloem sieve element during a feeding is a core part of plant–aphid interactions. Many of these proteins are aphid specific, and they have likely evolved to suppress plant defense and prevent occlusion of phloem sieve elements [76], which differentially localize in host cells and impact plant defenses.

The above information suggests that aphids can effectively evolve to mitigate plant defenses through the use of effector proteins; however, a more precise mechanism needs to be further elucidated, including the specific effectors in aphids and how they can be characterized.

## Sequestration of PSMs

**Figure 3:**
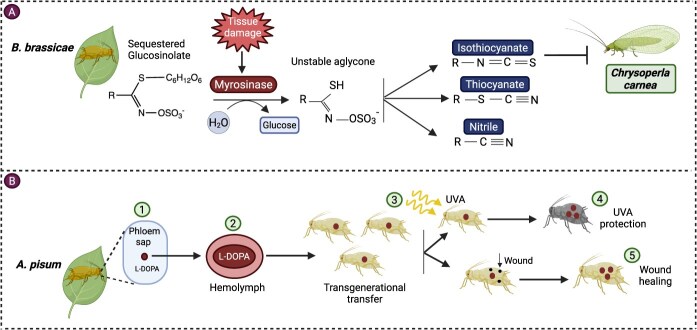
Mechanism of PSMs sequestration by aphids and their utilization in defense. (A) Aphids, such as *Brevicoryne brassicae*, sequester plant toxins like glucosinolates from Brassicaceae hosts and utilize them as part of their defensive mechanism against predators. When aphids are attacked by predators or upon tissue damage, these compounds are converted into an unstable aglycone in the presence of myrosinase by releasing a glucose molecule. This unstable aglycone spontaneously rearranges into toxic compounds, including conjugated isothiocyanate, which repels *Chrysoperla carnea*, as well as thiocyanate and nitrile [80]. (B) L-DOPA is considered a very toxic compound against many insects, but *A. pisum* sequesters L-DOPA from the phloem sap of *V. faba* and uses it for its defensive mechanism (1). (2) L-DOPA travels from the aphid’s gut to its hemolymph and was observed to transfer through generations (3). (4) *A. pisum* was observed to utilize sequestered L-DOPA for UVA protection. In an experiment involving the application of UVA radiation (360 nm), it was observed that L-DOPA undergoes a reaction, producing melanin pigments that cause aphids to darken and absorb UVA. (5) Similarly, L-DOPA-induced melanin was produced at wounded sites and sealed the wounds [77].

Sequestration is a biological process by which the insect herbivore ingests and retains plant defensive toxins in a position that allows it to acquire fitness. This complex form of adaptation, observed in approximately 250 insect species across six orders (Coleoptera, Heteroptera, Lepidoptera, Orthoptera, Hymenoptera, and Sternorrhyncha, also in Hemiptera like aphids), is based on the sequestration of metabolites from 40 plant families [77]. The phloem feeding results in the uptake of the plant’s defensive compounds by the aphids, including glucosinolates. Once ingested successfully, these compounds circulate throughout the insect’s body and are accumulated in the gut, or hemolymph, in either a free or conjugated form. For instance, glucosinolates sequestered by flea beetle *Phyllotreta armoraciae* are moved to the hemolymph from the gut; however, Sawfly larvae accumulate glucosinolates in the gut [78]. The mechanism, however, remains unexplained. Assumedly, specific proteins (ABC transporters) take part in their transportation across the cell membranes [79,80]. For example, the poplar leaf beetle (*Chrysomela populi*) has identified an ABC transporter, CpMRP, that is highly expressed in the defense glands and mediates the in vitro transfer of phenolglucoside salicin. Inhibition of CpMRP with RNA interference (RNAi) reduced the defensive secretion of the beetles, conclusively pointing out an important role played by this transporter in regulating defensive fluid synthesis [81]. Some insect herbivores also modify these compounds after sequestration to minimize their toxicity and enhance resistance properties, and use them as a defense against predators. The cabbage aphid *Brevicoryne brassicae* has developed an advanced sequestration mechanism, in which it selectively sequesters sinigrin (a glucosinolate) while actively secreting progoitrin (its structural analogue), making itself into a ‘walking mustard-oil bomb’ to deter predators [82]. For optimal anti-predator defenses, specific stable poisons must be triggered. *B. brassicae*, for example, generates a compartmentalized thio-glucosidase capable of cleaving the sequestered Glucosinolates, which emit isothiocyanates upon tissue damage [79]. *B. brassicae* has adapted to feed on selective aliphatic glucosinolates from Brassicaceae hosts and then activate them with its myrosinase enzyme. Laboratory evidence showed that the chemical defense of cabbage aphid predominantly produced less-toxic nitriles and conjugated isothiocyanates instead of single isothiocyanates, which are highly toxic, and still significantly affected the lacewing (*Chrysoperla carnea*). The partial detoxification response of *C. carnae* demonstrates that the aphid’s defense is still biologically effective [80] (Fig. 3A). The pea aphid has been observed to sequester a plant defensive metabolite, L-DOPA, by feeding on the host plant *Vicia faba*. L-DOPA (L-3,4-dihydroxyphenylalanine) is a highly toxic compound for some insects [77], including black soldier fly *Hermetia illucens* [83]*, Bombyx mori, Helicoverpa armigera*, *Drosophila* [84], and *Anopheles gambiae* [85]. In contrast, the pea aphid, unlike other aphid species, not only sequesters but also transmits it across generations and is observed to utilize it for protection against UVA radiation (360 nm) and wound healing by increasing the concentration of L-DOPA and melanin. This implies that there is a special adaptive advantage of chemical sequestration that is unrelated to classical defense roles [77] (Fig. 3B). On a spectrum of dietary specialization, the sequestration of cardenolides among four aphid species that feed on common milkweed (*Asclepias syriaca*) was minimal in the generalist *M. persicae*, increased in the highly specialized *A. asclepiadis* and *A. nerii*, and peaked in the monophagous *Myzocallis asclepiadis* [86]. Similarly, the specific aphid *A. nerii* sequesters the cardenolides of four milkweed hosts passively in a concentration-dependent manner but also seems to alter and concentrate some toxins not found in the plant. Interestingly, the sequestered cardenolides proved to be over-proportionately toxic as compared to those present in the host leaves, which in effect lessen predation by ladybird beetles despite the cost of growth. This study emphasizes that the trade-off between the costs and benefits of chemical sequestration depends on the quantities of toxins in plants and the presence of natural predators [87].

These examples demonstrate that aphids not only sequester PSMs but also utilize them as a benefit for their adaptation mechanism, creating a complex aphid–plant interaction that necessitates further molecular investigation of the molecular sequestration of defensive chemicals, which remains unclear, especially in aphids.

## Aphids’ transmitted virus-mediated adaptation

**Figure 4:**
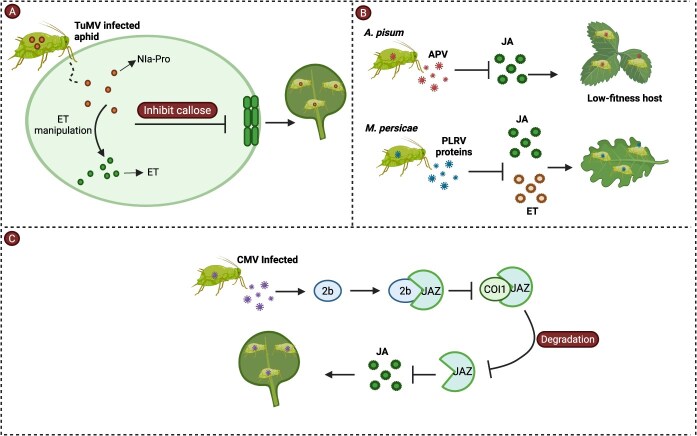
Mechanism of virus-mediated adaptation. (A) TuMV is transmitted by *M. persicae,* which manipulates the ET response in *A. thaliana* through the NIa-Pro protein. The NIa-Pro protein manipulates ET signaling and inhibits callose deposition, thereby increasing aphid survival and fecundity [92]. (B) APV is a symbiotic virus that is present in the gut and salivary glands of the aphid *A. pisum* and is horizontally transmitted to its host plants during feeding, suppressing JA signaling, which increases the aphid’s survival on low-fitness host plants, such as *V. villosa* [93]. Similarly, JA and ET responses induced by *M. persicae* are inhibited in *Solanum tuberosum* and *N. benthamiana* by PLRV proteins and facilitate the attraction and fecundity of aphids [94]. (C) CMV also manipulates host JA defense by directly binding its 2b protein to JAZ, which regulates JA signaling genes. The 2b protein weakens the functional binding between COI1 and JAZ, thereby preventing the degradation of JAZ and ultimately suppressing JA production in *Arabidopsis* [95].

Viruses transmitted by aphids badly affect the plant physiological system by manipulating or suppressing different defensive pathways of plants, including signaling pathways and the production of defensive PSMs. Almost all transmitted viruses negatively affect plants, promoting aphid adaptation and fecundity, and attract more aphids by causing the yellowing of host plants. For instance, *A.pisum*, which fed on pea plants infected with the bean leafroll virus (*Polerovirus genus*), exhibited a significant survival rate and enhanced fecundity relative to those grown on non-infected plants [88]. Research indicates that virus-infected yellow leaves primarily suppress repellent volatiles, such as cucumber mosaic virus (CMV), which suppresses α-copaene emission, ultimately increasing the vector aphid attraction [89,90]. Barley yellow dwarf virus infects wheat plants and causes yellowing of its leaves and stunted growth by the activities of virus-encoded small interfering RNAs (vsiRNAs). An example is VSI RNA8856, which has been reported to silence a chlorophyll synthase gene, thereby disrupting chlorophyll biosynthesis [91]. Generally, aphid-transmitted viruses promote aphid adaptation to the host plant in two ways, including modulating signaling pathways and altering VOCs emission. In case of disrupting the signaling pathways in the host plant, the infection leads to increased fecundity and virus spread, such as Turnip mosaic virus (TuMV), which manipulates ET response in *A. thaliana* by the action of its nuclear inclusion a-protease domain (NIa-Pro protein), which blocks callose formation when infected by aphids, thereby increasing the fecundity of *M. persicae* and weakening the plant’s defense [92] (Fig. 4A). Acyrthosiphon pisum virus (APV) is a nonreplicating symbiont virus that has been found in the salivary gland and the guts of *A. pisum* and is horizontally transmitted to the host plant during feeding and can suppress the JA signaling pathway. Such repression results in increased aphid survival on low-fitness host plants, including *V. villosa* [93] (Fig. 4B). The potato leafroll virus (PLRV) suppresses JA and ET responses induced by aphids in *N. benthamiana* and *Solanum tuberosum*, promoting aphid recruitment and fecundity. This suppression in the plant-aphid association was identified to be mediated by three PLRV proteins (P0, P1, and P7). Such findings explain the influence of circulative viruses in blocking the defense of the host plant, thus playing an advantageous role in the colonization process of vectors and the spread of the virus [94] (Fig. 4B). In a similar way, CMV utilizes the host JA defense system by binding jasmonate ZIM-domain proteins (JAZ), which by default have inhibitory effects on JA-responsive genes. The 2b protein of CMV directly interacts with the JAZ protein, weakening the interaction of COI1 and JAZ and avoiding its degradation, which finally represses the generation of JA in *Arabidopsis* [95,96] (Fig. 4C).

Similarly, plant viruses also manipulate VOCs secreted by host plants by altering the attractive or repellent patterns towards aphids, which in turn may result in increased attractiveness of these vectors to virus-infected plants. Different experiments have shown that infected plants, when exposed to viruses, produce a variety of VOCs, whereas healthy plants do not. These deformed VOCs can attract aphids. As a typical example, the alteration of VOCs in tomato plants infected with the Fny strain of CMV attracts specialist aphids, *M. persicae*, and generalist aphids, *Macrosiphum euphorbiae* [97]. Additionally, infected banana plants emit more VOCs in response to banana bunchy top virus infection, making them more attractive to *Pentalonia nigronervosa* aphids [98]. Bean common mosaic virus (BCMV), common bean mosaic necrosis virus (BCMNV), and CMV also stimulate aphid movement as a result of surface cues and a blend of volatiles [89]. Plants release numerous VOCs, including aliphatics, terpenes, phenylpropanoids/benzenoids, and other compounds in response to herbivore stress. For instance, sesquiterpene and salicylate emissions are the ultimate indication of aphid attack on Scots pine [99]. Apart from defence mechanisms, some volatile semiochemicals in the host finding process, independently of visual stimuli, in Aphids [100]. The production of terpenoid compounds is suppressed in tobacco plants infected with tomato yellow leaf curl virus, which lowers the plants’ resistance to aphids. *Cucurbita pepo* plants infected with CMV exhibit enhanced production of VOCs, hence rendering the plants more appealing to aphids. VOCs direct aphids toward infected plants, enabling them to acquire the virus while feeding. Subsequently, aphids that have fed on CMV-infected plants search for other hosts and spread the virus to non-infected plants [16].

In short, this complex interaction between aphids and viruses, whether directly or indirectly, weakens the plant’s defense, attracts more virus vectors, facilitates the proliferation of both aphids and viruses, and warrants further molecular investigation.

## Behavioral adaptation

Aphids possess well-developed behavioral and physiological mechanisms to counteract the PSMs that serve as plant defenses. Insects may employ various cues in their feeding decisions, with chemical cues, such as volatile compounds detected before feeding or compounds identified during the initiation of feeding, often playing a significant role [101]. Insect herbivores select multiple hosts for nutrition, favoring plants with lower concentrations of secondary metabolites [102]. The feeding site of aphid populations on a specific host plant is also influenced by the nutritional and secondary metabolite composition present in the phloem sap [103]. When selecting hosts, aphids probe them with their stylets to evaluate whether the plant material contains sufficient PSMs (e.g. glucosinolates or alkaloids) in the mesophyll tissues; aphids frequently retract their stylets when they detect a toxic compound to prevent ingestion. Molecular analysis has shown that the calcium-binding proteins present in saliva are actively involved in inhibiting initial defense reactions in the plant, such as calcium bursts of ion signaling initiated by stylet penetration, thereby limiting the callose deposition and phloem occlusion. For instance, *A. gossypii* can avoid watermelon varieties with increased levels of ROSs and significant callose deposition in phloem elements, such as JinMeiDu, compared to sensitive varieties like XiNong [104, 105]. Aphids indicate specific host selection in response to different plant defensive compounds. For example, Aphids showed a preference for feeding on Chinese cabbage (*Brassica rapa pekinensis*) over radish and cabbage (*B. oleracea*), attributed to the reduced concentration of sulfur-containing phytochemical secondary metabolites, specifically glucosinolates, and the elevated amino acid content in the leaves [106]. Similarly, the host-adapted biotypes of *A. pisum* exhibit different feeding efficiencies on various genotypes of peas. Pea-adapted biotypes ingest phloem sap up to three times more rapidly on *P. sativum* than alfalfa-adapted biotypes, suggesting optimization of behavioral probing specific to the host plant [107]. Haplotypes of *A. gossypii* Hap3 and Hap17 showed discrete host preferences on the *Capsicum annum* varieties, with Hap3 exhibiting the best performance on Saierweilvtianjiao and Lvzhou101, while Hap17 preferred Lvzhou101 [108]. Alfalfa-specialized pea aphids show tolerance to flavonoids and saponins in *Medicago sativa* through adaptations. In contrast, clover-adapted aphids perform poorly on alfalfa, suggesting a form of host race-specific detoxification capacity [109].

In short, this information suggests strong evidence that aphids avoid cultivars with increased plant defense and exhibit adaptive host-shifting behavior. In contrast, direct evidence of adaptation in different plant feeding site selection, including younger versus older leaves or plant tissues, remains unclear. Similarly, crowding stress in aphid populations triggers wing formation [110]; however, no specific evidence has been confirmed yet to suggest that PSMs can directly trigger alate development. These unexplored behavioral adaptations of aphids indicate crucial areas for future study.

## Aphid-resistant molecular plant breeding as prospect and future directions

Breeding new horticultural crop varieties that naturally produce a wide range of PSMs could provide a sustainable strategy for reducing aphid damage. Advancements in molecular plant breeding have led to extremely accessible approaches that are both swift and accurate. Researchers and plant breeders can expedite the molecular development of aphid-resistant plant varieties by integrating population genetic analysis, multi-omics methodologies, and gene-editing technologies such as CRISPR/Cas9. The use of wild relatives and traditional landraces has successfully introduced defense compounds, such as glucosinolates and phenolics, into contemporary crop lines. Methodologies such as GWAS, QTL mapping, and BSA (bulked segregant analysis) are essential tools for elucidating the complicated genetic architecture of intricate traits. These tools facilitate the identification of potential genes associated with plant resistance to aphids. Genetic linkage maps have been constructed using diverse markers, including simple sequence repeats (SSR), amplified fragment length polymorphisms (AFLP), and single-nucleotide polymorphisms (SNPs), which have led to the identification of QTLs for aphid resistance. Such investigations have been conducted on a diverse array of plants, including significant crops such as maize, wheat, cucumbers, soybeans, and others [16]. Brassica crops have acquired aphid resistance from their wild relatives. For instance, resistance from *B. fruticulosa* was successfully moved into *B. juncea*, which led to stable backcross lines. Although PSMs were not directly quantified, a high concentration of glucosinolates in the wild donor is indeed indicative of a PSM-mediated process, a fact that supports the feasibility of QTL mapping [111]. Similarly, the acylsugar-enriched lineage derived from *Solanum pennellii*, which conferred resistance to whitefly [112]. In cotton, the introgression of hybridization between the aphid-sensitive Xinluzao 50 cultivar and the aphid-resistant Xinluzao cultivar population-based QTL mapping in the F2 generation evaluated GhLAC4-3, which encodes an enzyme associated with lignin biosynthesis and aphid sensitivity. Further studies on virus-induced gene silencing have supported that this gene plays a positive role in regulating aphid resistance in cotton [113]. Moreover, the GhCalS5 gene was shown to increase cotton resistance to aphids by stimulating the deposition of callose, which hinders phloem sap intake, enhances aphid feeding, and inhibits growth and reproductive processes [75]. The combination of SSR and SNP markers for genotyping has been successful in genetic mapping of the *A. craccivora* resistance locus in the cowpea breeding line SARC 1-57-2 [114]. The development of high-throughput sequencing has enabled the broader application of GWAS and BSA procedures in the research and discovery of plant aphid resistance genes. GWAS have identified the existence of sequence differences, termed SNPs, in the plant genome, and have generated SNPs associated with aphid resistance characteristics. GWAS is a method that has been effective in recent genomics studies for evaluating the genetic and biochemical mechanisms of various plant metabolic pathways. For example, the flavonoid pathways in wheat and the phenylpropane metabolic pathway in barley were elucidated using mGWAS; specifically, flavonoid-O-glucoside was identified as a resistance factor against *Spodoptera litura* [115]. A GWAS on *P. sativum* resistance to both a pea-adapted and a nonadapted biotype of *A. pisum* identified a significant genomic region on chromosome seven associated with resistance to both biotypes. This region, recognized as the major-effect QTL *ApRVII*, is linked to substantial linkage disequilibrium blocks that correlate with resistance to either or both aphid biotypes [116]. BSA has demonstrated effectiveness in identifying aphid resistance traits by selecting parent individuals that exhibit distinct phenotypic extremes for breeding purposes. In peaches, mapping identified the Rm3 locus on chromosome 1, localized to a 160-kilobase region that contains 21 genes [117].

Meanwhile, modern genome editing techniques, such as CRISPR/Cas9, emphasize crop resistance against insect pests through various means, including genome editing in bacteria to combat plant viruses. For example, CRISPR was used effectively against Gemini viruses, including Beet curly top virus, Tomato yellow leaf curl virus, and Merremia mosaic virus, to protect crop plants that directly or indirectly manipulate plant defenses and prefer insect attractiveness and adaptation [118]. The CRISPR technique has some limitations in the case of aphids, as it takes a considerable amount of time to knockout a single gene. Due to aphids’ complex reproductive abilities, they often fail to yield promising and consistent knockdown results when injected or fed RNAi molecules. For instance, the technique Symbiont-mediated RNAi was tested to engineer *Serratia symbiotica* in *A. pisum*, which failed to silence the salivary effector protein gene C002 reliably. However, this technique has been successfully applied in other insects, including kissing bugs, thrips, and honeybees [119]. Therefore, the focus should be on plant-based genetic engineering. Tactics such as editing resistance genes through CRISPR, HIGS, and introgression of PSM resistance characteristics from wild relatives can be an alternative solution to creating aphid-resistant crops. *VST1* is a negative regulator of aphid resistance in watermelon; CRISPR-induced silencing of *VST1* decreased aphid attraction, whereas overexpression made watermelons more susceptible to aphid infestation [120].

However, aphid effectors and virus transmission can be countered by *Vat* genes, specifically the Coiled-Coil NLR (CC-NLR) class, which recognizes aphid saliva, thereby triggering callose/lignin defense and inducing ETI. *Vat-1^PI 161 375^*, a CC-NLR gene in a well-diversified NLR cluster of melon, provides resistance to *A. gossypii* and subsequent virus transmission [121,122]. Similarly, the Vat gene in melon confers resistance by reducing the colonization of *A. gossypii* and the transmission of related viruses, such as CMV and cucurbit aphid-borne yellows virus. [123]. Meanwhile, other genes, such as rag genes (*Rag1* and *Rag2*), conferred resistance against the soybean aphid by rapidly inducing lignin deposition, phenylpropanoids, and cell-wall reinforcement, showing stronger resistance than either single gene [124]. According to Yang *et al.* [125], a new *RagFMD* gene was identified in the soybean landrace Fangzheng Moshidou, which provides robust resistance to aphids even under high pest pressure.

As aphids have an array of behavioral adaptations to evade plant defensive strategies, plants also employ molecular interaction-based defense signaling and breeding-based innovations to respond to such adaptations. Plants have a complex signaling network of hormones that control plant defenses against insects. The accumulation of indole-3-acetic acid (IAA) is rapidly caused by herbivory, which, within minutes, has a systemic distribution and is followed by the burst of JA [126]. In chickpea, oral reception of *Helicoverpa armigera* induced JA/ethylene signaling within 20 min and repressed growth-related pathways, including auxin and gibberellin, and this model indicated fast transcriptional rewiring of hormone networks [127]. On a bigger scale, the three pathways (JA, SA, and abscisic acid (ABA)) converging to defense gene expression, antioxidant reactions, and secondary metabolite synthesis are combined to constitute plant immunity [128]. Hormone crosstalk between JA, SA, ABA, and IAA was observed in lentil landraces subjected to *A. pisum*, and reprogramming of alkaloid, phenolic, flavonoid, and fatty acid metabolism occurred [129]. These observations highlight the importance of hormone signaling in controlling the aphid-induced defense responses and also in causing metabolomic changes to produce PSMs as important breeding targets. Hormone-responsive characteristics and accumulation of metabolites in the breeding programs can be of great benefit to develop aphid-resistant cultivars as a result of integration.

Molecular plant breeding has achieved considerable success in enhancing plant resistance to aphids. There is continuous improvement in molecular technologies for plant breeding, which offer a broad scope of possibilities to improve plant resistance to aphids. Population genetics, combined with sophisticated techniques and multiomics methodologies like CRISPR-Cas9, is enabling scientists and breeders to develop new plant varieties that are resistant to insects. Other valuable tools include GWAS, QTL, and BSA mapping, as well as the identification of key genes for aphid resistance in plants. The molecular mapping used to generate genetic bacterial linkage maps enables the mapping of resistance loci at the correct locations. The deployment of *Vat*-like NLR genes is a practical means of achieving broad-spectrum resistance in crops against insects, such as the aphid, and the virus diseases they transmit, in future breeding programs for crops. In addition, possibilities exist for introducing genes through genetic engineering procedures, which allow plants to synthesize defensive PSMs harmful to aphids, thereby reducing their attraction, or modifying the aphids’ attraction, thereby strengthening their innate defense mechanism. This creates more resilient plant varieties. All these have been used to produce new lines that are more resistant to aphids in several crop species.

## Conclusion

This review examines how aphids interact with PSMs and summarizes evidence that PSMs can limit herbivory. Remarkably, aphids overcome toxic chemicals using a diverse array of resistance tools. Their arsenal includes cytochrome P450 enzymes, GSTs, and carboxylesterases that chemically detoxify compounds, superoxide dismutase and catalase that protect against oxidative damage, and ATP-binding-cassette transporters that export plant toxins. Endosymbiotic bacteria, particularly *Hamiltonella defensa* and *S. symbiotica*, enhance this resistance mechanism by modulating the plant’s defense responses. Aphids also deploy salivary proteins that neutralize defensive signals, sequester toxins for their defense or to repel predators, and indirectly promote the spread of harmful viruses, which further reduce plant resistance. Behaviorally, they probe selectively, shift among hosts, or feed on lower-PSM tissues to minimize exposure. Given this ongoing arms race, durable management of aphid pests will need to extend beyond the use of broad-spectrum insecticides and develop aphid-resistant cultivars with elevated or precisely tuned PSM profiles. Molecular breeding methods can be used to introduce resistant traits from wild resources to original cultivars. Recent advances in molecular breeding tools, including high-resolution genome mapping and targeted functional gene discovery, have substantially accelerated the development of aphid-resistant crop varieties. Combined with knowledge of aphid evolution, such targeted PSM manipulation could lead to sustainable, long-lasting resistance in agroecosystems worldwide.
